# Bulletin of the Public Health

**Published:** 1879-04

**Authors:** J. B. Hamilton

**Affiliations:** Acting Surgeon-General U. S. Marine Hospital Service


### BULLETIN OF THE PUBLIC HEALTH,

Issued from the Office of the Surgeon-General U. S. Marine Hospital Service, under the National Quarantine Act of 1878.

No. WEEK ENDED MARCH 22, 1879. 37.

| CITIES. | Total Deaths. | Annual rate per 1000 of Popul'n. | DEATHS FROM Diph-theria. | Scarlet Fever. | Enteric Fever. | Acute Lung Disease | Phthisis |
|---|---|---|---|---|---|---|---|
| New York* | 623 | 30. 6 | 14 | 65 | 1 | 113 | 89 |
| Philadelphia | 278 | 16. 7 | 7 | 12 | 4 | 34 | 48 |
| | | | 37 cases | 96 cases. | | | |
| Brooklyn | 208 | 19.16 | 12 | 8 | 2 | 37 | 25 |
| St. Louis | 114 | 12 | 2 | ... | 2 | 13 | 20 |
| Chicago | 138 | 15. 6 | 12 | 4 | 4 | 24 | 12 |
| | | | 14 cases | 13 cases. | | | |
| Boston | 147 | 21 | 8 | 3 | 1 | 21 | 23 |
| Baltimore* | 147 | 21 | 4 | 6 | 1 | 28 | 26 |
| Cincinnati | 116 | 21. 5 | 6 | 18 | 2 | 20 | 10 |
| Dist. of Columbia* | 77 | 25 | 1 | 5 | 1 | 29 | 12 |
| Pittsburg | 42 | 15 | 4 | 3 | 2 | 7 | 3 |
| Buffalo* | 35 | 13 | 6 | 4 | ... | 1 | 6 |
| Cleveland | 51 | 16. 4 | 2 | 1 | ... | 11 | 4 |
| Newark | 67 | 27. 9 | 3 | 2 | 2 | 6 | 15 |
| Providence* | 30 | 15. 6 | 1 | ... | 1 | 4 | 8 |
| Richmond..* | 25 | 16.45 | ... | 2 | ... | 3 | 4 |
| New Haven | 26 | 22. 5 | 1 | 1 | ... | ... | ... |
| Portland, Me | 13 | 19 | 1 | ... | ... | 1 | 3 |
| Savannah* | 17 | 31. 5 | ... | ... | ... | 2 | 4 |
| Total for Week | 2154 | Average. 20. 2 | 84 | 134 | 23 | 352 | 312 |
| San Francisco* Week ended March 14. | 83 | 14. 4 | 5 | 1 | 1 | 11 | 10 |
| New Orleans* Week ended March 16. | 84 | 20. 8 | 1 | ... | ... | 18 | 17 |
| Montreal* Week ended March 15. | 71 | 31 | 12 | ... | 1 | ... | 7 |

*REMARKS.—New York—Whooping Cough caused 26 deaths. Baltimore—Measles 2; Whooping Cough 8; Cerebro Spinal Fever 2; Erysipelas 2. Dist. of Columbia—Pneumonia and Infantile Convulsions prevalent. Buffalo—Cerebro Spinal Fever 3; Croup 4; Erysipelas 1. Providence—Public health better than usual at this season. Richmond—Whooping Cough 1. Savannah—Whites, 7 deaths; colored 10. San Francisco—Cerebro Spinal Fever 2; Croup 1. New Orleans—Diseases of air passages prevalent. Montreal—Small Pox 12.

BERMUDA.—In a population of 15,300, there was but *one* death (from old age) during the two weeks ending March 11th. Two cases of yellow fever in hospital.

HAVANA.—Week ended March 22d. Small pox caused 11 deaths; yellow fever 1. During the month of February the total deaths were 632—an annual rate of 42 per 1,000 of the population. Diarrhœal diseases caused 70 deaths, small pox 45, yellow fever 13, malarial fever 16, enteric fever 10, diphtheria 7.

Great Britain.—Week ended March 8th. In the 23 large cities the average death-rate was 29 per 1,000. Rate in Oldham, 43; Dublin, 36; Manchester, 33; Liverpool, 30; London, 29; Sheffield, 29; Glasgow, 21; Edinburgh, 19; Plymouth, 19. Small pox caused 17 deaths in London, 11 Dublin. Scarlet fever was very prevalent, and measles excessively fatal in several towns.

Paris.—Week ended March 6th; 1,060 deaths. Rate 27. Enteric fever caused 29 deaths.

German Empire.—Week ended March 1st. In 149 towns the average death-rate was 28.1 per 1,000. Rate in Munich, 34; Strasburg, 34; Dresden, 30; Breslau, 28; Cologne, 28; Hamburg, 26; Berlin, 25.7; Frankfort, 23. The whole number of deaths were 4,093, of which measles caused 62, scarlet fever 62, diphtheria 161, whooping cough 63, enteric fever 61, phthisis 590, acute lung diseases 506.

Vienna.—Week ended March 1st; deaths 438. Rate 31. Small pox caused 6 deaths.

St. Petersburg.—Week ended February 22d; deaths 620; Rate 48. Small pox caused 56 deaths, fevers 49. The imperial order directing the burning of the infected houses at Wetlyanka has been executed in part, and extraordinary powers have been conferred on the Governor of the district, under which vigorous efforts are being made to improve its sanitary condition. The towns adjoining the infected district, as well as the cities of St. Petersburg and Moscow, are taking measures against the possible extension of the disease on the approach of warm weather, by cleansing and disinfection of unsanitary quarters, issuing of cooked food to the poor, erection of temporary buildings for the reception of refugees from the infected districts, and furnaces for burning infected clothing.

J. B. Hamilton,

*Acting Surgeon-General U. S. Marine Hospital Service.*

